# Overlooking the obvious: the importance of communicating safety and risks of opioids-the Israeli context

**DOI:** 10.3389/fphar.2024.1356968

**Published:** 2024-05-24

**Authors:** Maram Khazen, Shelly Kamin-Friedman

**Affiliations:** ^1^ Max Stern Academic College of Emek Yezreel, Emek Yezreel, Israel; ^2^ Department of Health Policy and Management, School of Public Health, Faculty of Health Sciences, Ben-Gurion University of the Negev, Beer-Sheva, Israel; ^3^ Department of Bioethics, The School of Public Health, Faculty of Social Welfare and Health Sciences, University of Haifa, Haifa, Israel

**Keywords:** patient-clinician communication, opioid epidemic, risk and harms communication, difficult clinician-patient conversations, legal aspects in communicating opioid risks, strategies to mitigate opioid risks

## Introduction

The opioid crisis has been defined as a patient safety problem, “the avoidance, prevention, and amelioration of adverse outcomes or injuries stemming from the process of healthcare” (Vincent, 2006). Opioid use might lead to side effects (e.g., respiratory depression, confusion), and its abuse can result in developing high tolerance levels and increasing dosage beyond the prescription level (opioid use disorder- OUD), causing mortality and morbidity (Miron et al., 2022).

In Israel, high consumption rates of prescription opioids have been reported (Dressler et al., 2020), wherein by 2020, it was placed first worldwide (International Narcotics Control Board, 2020). Studies of prescription opioid procurement point to high fentanyl consumptions (50 times more potent than Heroin) by the younger Israeli population and non-malignant patients (Miron et al., 2021) in outpatient settings (Shapira et al., 2023). These consumptions may have not concluded with high mortality rates due to mitigation of OUD cases and misclassification of death causes related to opioid (Shapira and Rosca, 2022). Regardless, they point to a public health crisis that needs to be addressed (Davidovich et al., 2022).

Pharmaceutical companies and the possession of illegal non-prescribed synthetic opioids have been suggested as main contributors to the crisis (Gomes et al., 2018; Hadland et al., 2019). A less examined aspect in the literature relates to conversations about risks and how clinicians communicate to patients about opioid safety. Despite Israel’s high opioid prescription rate, no formal regulations emphasize communication about opioid risks and safety as key to managing opioids consumption. The ministry of health calls for reducing opioid prescriptions, tapering high doses, and identifying patients in risk. However, it does not stimulate provider communication as integral to addressing opioid addiction and overdose (The Ministry of Health, 2022).

This opinion piece conjures that managing the opioid crisis is entrenched in how and why clinicians communicate opioid risks, particularly, to non-terminal patients experiencing chronic pains. It does not examine the appropriateness of prescribing opioids for chronic pains, rather, best ways to communicate risks when prescribing opioids.

## Breaking down the meaning of good communication: the why and the how

Communicating opioid risks and safety during the clinical encounter entails clinicians acknowledging why it is important to provide an explanation and how to communicate this explanation to patients. This is important since some clinicians do not perceive this type of communication as part of their job (Shields et al., 2018).

### Why communicate risks and safety

Providing medical information and its impact on pain outcomes and opioid use, have been suggested in ample studies (Rudd et al., 2016; Thakur et al., 2021). Usually, Clinicians provide information limited to common side effects and adverse drug reactions, leaving out opioid dependency and addiction risks (Thakur and Chewning, 2020). A missed opportunity to communicate opioid risks to patients might lead to even higher consumption rates and to legal implications. According to the Israeli law (Section 13 of the Patient’s Rights Law, 1996) “informed consent” is required prior to all medical treatments. To obtain “informed consent,” clinicians must provide information about potential risks to enable patients to make an informed decision. As stipulated by Israeli court judgements, patients have the right to receive information about treatment risks (Civil Case, 2019), considering clinicians’ advantage in medical knowledge and experience (Civil Appeal, 2019b).

The requirement for “informed consent” is based on the right to autonomy and freedom of choice: Individuals have the right to decide and act according to their needs and what they see appropriate. Therefore, withholding information from patients violates the right to autonomy. According to the Israeli court judgements, patients should be compensated, if they experience risks of a treatment and can establish that they would have avoided the treatment had they been informed of the risks (Civil Appeal, 2019a).

### How to communicate risks and safety issues

In 2018, the Israeli Ministry of Health published an “information sheet” regarding opioid addiction risks (The Ministry of Health - Division of Pharmaceuticals, 2018). An addicted person was defined as: “Person suffering from addiction spends most of his day in a compulsive search for the drug, neglecting his work, studies, leisure time, and family. Also, the addicted person develops different ways to obtain the drug when it is not available. Abstinence symptoms may appear when he/she fails.” The “information sheet” further noted a hereditary tendency for addiction, The sheet further holds instructions for avoiding opioid overdose and abuse and consulting the clinician regularly. Clinicians may distribute the “information sheet” to patients when prescribing opioids. However, handing out an “information sheet” does not ensure the patient understands the information, nor conversed with the clinician about risks and treatment alternatives.

Clinicians should be guided by best practices to provide information about opioid risks in a thoughtful and transparent manner, while being respectful of the patient’s pain. However, to do so, they need support systems and strategies instigated by patient safety ideals, suggested as follows.

#### Requiring a written consent form prior to opioid therapy

A consent form for medical treatments specifies the risks and opioids’ side effects and OUDs. Having patients sign a consent form prior to initiating a treatment might ensure that they understand treatment goals and risks. The importance of signing a consent form underlies in triggering a patient-clinician conversation. Hence, if initiating an opioid treatment requires signing a consent form, it will probably lead to discussing risks (Cheatle and Savage, 2012).

#### Signing an opioid therapy agreement

An agreement signed by patients and clinicians can also initiate discussions and information provision. In addition to aspects emphasized in the some opioid agreements (e.g., single prescribers and specified designated pharmacy; pill counts, random urine screens, and regular follow-ups), it is imperative that this type of agreement includes treatment goals and risks, patient’s and clinician’s responsibilities. Specifically, the opioid agreement should address the treatment rationale and plan (e.g., contingencies, expected improvement, time frames). Clinicians’ responsibilities may include seeing the patient within a reasonable time for follow-ups and discussing with him/her urine testing results before making decisions about future treatments. Also, Clinicians are obligated to receive updates about pain treatments including opioids. The agreement will state terms for opioid discontinuation, such as consuming illegal substances while taking an opioid or sharing the opioid medication with others (Manchikanti et al., 2017).

#### Providing communication tools to patients

Introducing patients to communication tools has been shown to raise awareness and help clinicians to provide information relevant to opioids (Meisel et al., 2022). These tools include narrative and illustrative stories about experiences of other patients and probabilistic risk information (Meisel et al., 2022). Some tools comprise an individualized risk assessment of opioid misuse, while others hold narratives integrated in decision aids to facilitate the decision to taper opioid use (Dolan et al., 2022). The persuasive narratives are mostly either based on a highly engaging story (Henry et al., 2021) or developed around the role of storytellers (Stone et al., 2021).

Further, communication tools can focus on alternative therapies after surgeries to minimize opioid use and dependence (Campbell et al., 2019).

#### Assuring culturally and linguistically accessible information

Low-income, linguistic, and cultural minority populations with limited digital skills might experience barriers to accessing relevant information (Jackson et al., 2021). For healthcare systems to support these patients, it is pivotal to produce and disseminate information culturally competent and approachable to diverse populations. This information should focus on preserving one’s autonomy and being informed about treatment options, risks, and safety issues. Hence, clinicians, written consent forms, and opioid therapy agreements, need to be diligent of patients’ cultural background.

### Addressing difficult clinician-patient conversations regarding opioid prescribing

Clinician-patient conversations regarding opioid prescribing might prove to be challenging. Particularly if patients are in pain and have experienced unsuccessful treatments with non-opioid medications or if the diagnostic assessment of the pain is fraught with uncertainty. Thus, a best-practices guide might benefit clinicians who prefer to prescribe alternatives to opioids or need to explain to patients about opioid risks. Points to emphasize in the guide based on literature (Opioid Therapy for Chronic Pain Work Group, 2017; Hulen et al., 2018; Wyse et al., 2019) are in three stages. First stage includes validation of patients experience and symptoms (e.g., acknowledge the impact of pain symptoms, understand the patient’s everyday social context, and refrain from using stigmatizing words when referring to opioid consumers such as “abusers” and “addicts” (Goodyear et al., 2018). Second stage, a detailed deliberation about harms and potential risks of opioids. The risk deliberation might highlight that there is no safe dose of opioids, risk increases with dose, risk of patient attachment to opioids might lead to confrontations and impact patient-provider communication and care delivery. The guide can conclude with a concrete treatment plan (e.g., setting expectations regarding pain management and reevaluation in 3 months, close monitoring of opioid consumption) and emphasize alternative strategies to alleviate pain.

#### Engaging pharmacists in efforts to communicate opioid risks

Pharmacists are the most accessible healthcare providers and key figures in promoting patient safety. In Israel, their relationship with patients is often characterized as close, long-term, and sometimes based on friendships (Khazen and Guttman, 2022). Hence, they are integral to mitigating the mortality and morbidity of the opioid crisis. They can prevent opioid misuse by screening and monitoring opioid prescribing and use [e.g., an electronic database Prescription Drug Monitoring Programs (Holmgren et al., 2020)], providing access to naloxone (an opioid antagonist, used to reverse/reduce opioids’ effects), assisting in the rehabilitation process of patients with an OUD, and ensure a continuous care delivery for patients in pain (Bratberg et al., 2020). Based on their relationship with patients, pharmacists can also support and advise clinicians contemplating whether to provide an opioid prescription.

## Discussion

### Where do we go from here?

Communicating about opioids risks to patients at the onset of the treatment is pivotal. This is in light of studies emphasizing challenges of opioid tapering (McNeilage et al., 2021) and those suggesting that patients who discontinue prescribed opioids are more likely to begin consuming non-prescribed opioids (Binswanger et al., 2020; Coffin et al., 2020).

Examining the why and how to break down the meaning of good communication about opioids between clinicians and patients should be stressed in the guidelines of opioid prescribing. Also, legal and structural changes are advised.

#### Legal change

Regulation anchoring is required for advocacy strategies that obligate patients and clinicians to sign a consent form or an opioid therapy agreement before receiving a treatment. The current Israeli Patient’s Rights Law, 1996, requires signing a consent form prior to specific procedures (surgeries, catheterizations, dialysis, radiotherapy, IVF, and chemotherapy). An opioid treatment written consent would require legislative changes to become part of the treatment procedure.

#### The work environment

Studies point to clinicians as main players regarding communicating opioid risks to patients. However, how well clinicians communicate with patients during medical encounters has long been questioned (Daniels et al., 2012). Failure to communicate opioid risks might be attributed to workloads and maintaining the workflow, when clinicians feel beleaguered in their jobs as is, leaving less room to converse with patients. A hectic work environment and heavy workloads can hinder the communication process, add to clinician burnout, and result in less attentiveness to pain experienced by the patient (Khazen et al., 2023). Thus, healthcare systems should advance regulations for additional allotted time during clinical visits that involve opioid prescribing. Additionally, there is a need for a quality control system that incentivizes clinicians to follow up on patients that were prescribed opioids by sending out reminders or having a designated staff member follow up on these patients and schedule appointments with their clinician.

#### Engaging pharmacists as second-primary care physicians

Studies allude to the need to improve pharmacists’ process of screening patients. According to Israeli Ministry of Health guidelines, pharmacists must inform patients of addiction risks when supplying opioids. They are required to refer patients to the 2018 “information sheet” (Opioids, 2022). Merely referring patients to an “information sheet” might not suffice. It is advised that pharmacists’ role in Israel be redefined and extended—from a medication dispenser to a second-primary care provider. In addition to the traditional tasks, the new role would include screening patients who show OUD and referring them to their clinician or contacting the clinician on their behalf and recommending alternative treatments (e.g., NSAIDs, non-pharmacological therapies), and monitoring these patients by initiating follow-ups within regular time intervals (Farrell et al., 2013). This new role can be facilitated by trust and respect characterizing the personal patient-pharmacist relationship (Khazen and Guttman, 2021; Khazen and Guttman, 2022).

## Conclusion

The great increase in opioid prescribing in Israel for non-terminal chronic pain patients calls for integrating communicational strategies in managing the opioid crisis. These strategies can aim to improve clinicians’ communication skills to discuss risks with patients in a linguistically and culturally accessible manner. Further, structural changes of additional allotted time for opioid prescribing sessions and integrating the pharmacists in the process of improving patient safety, can prove to be beneficial.

Communication skills are essential for improving patient safety, providing high-quality care, and mitigating the opioid crisis. These skills need to be integrated into medical training to support healthcare providers when initiating opioid prescribing, monitoring and screening patients, and during difficult conversations with patients with OUD. A less transparent communication process might make patients feel that their pain is disregarded causing them to inflate symptoms and pain, withhold information such as family-history addiction or a higher opioid dosage consumption. Acting only to limit opioid prescribing without promoting risk communication will not suffice. While it may minimize the number of prescriptions, illegal ways of obtaining these medications might emerge, augmenting the opioid crisis and resulting in no more than a pyrrhic victory.
